# Keeping it real: Looking beyond capacity limits in visual cognition

**DOI:** 10.3758/s13414-021-02256-7

**Published:** 2021-03-31

**Authors:** Árni Kristjánsson, Dejan Draschkow

**Affiliations:** 1 School of Health Sciences, University of Iceland, Reykjavík, Iceland; 2 School of Psychology, National Research University Higher School of Economics, Moscow, Russia; 3 Oxford Centre for Human Brain Activity, Wellcome Centre for Integrative Neuroimaging, Department of Psychiatry, University of Oxford, Oxford, UK

**Keywords:** Visual attention, Visual working memory, Visual long-term memory, Virtual reality

## Abstract

Research within visual cognition has made tremendous strides in uncovering the basic operating characteristics of the visual system by reducing the complexity of natural vision to artificial but well-controlled experimental tasks and stimuli. This reductionist approach has for example been used to assess the basic limitations of visual attention, visual working memory (VWM) capacity, and the fidelity of visual long-term memory (VLTM). The assessment of these limits is usually made in a *pure* sense, irrespective of goals, actions, and priors. While it is important to map out the bottlenecks our visual system faces, we focus here on selected examples of how such limitations can be overcome. Recent findings suggest that during more natural tasks, capacity may be higher than reductionist research suggests and that separable systems subserve different actions, such as reaching and looking, which might provide important insights about how *pure* attentional or memory limitations could be circumvented. We also review evidence suggesting that the closer we get to naturalistic behavior, the more we encounter implicit learning mechanisms that operate “for free” and “on the fly.” These mechanisms provide a surprisingly rich visual experience, which can support capacity-limited systems. We speculate whether natural tasks may yield different estimates of the limitations of VWM, VLTM, and attention, and propose that capacity measurements should also pass the real-world test within naturalistic frameworks. Our review highlights various approaches for this and suggests that our understanding of visual cognition will benefit from incorporating the complexities of real-world cognition in experimental approaches.

## Introduction

Lecturers in visual perception and cognitive psychology often wow undergraduates by showing them well-designed experiments that highlight the limitations of various aspects of visual cognition. How inefficient they are when searching for the yellow vertical bar amongst an array of yellow horizontals and blue verticals and how half the class misses a turbine engine disappearing and reappearing in plain sight. The students are surprised because these examples strongly contrast with their experience of being efficient visual searchers and never missing the lack of a critical plane component in their visual field. But natural behavior in natural environments is so seamless because properties of the visual system enable us to overcome all these limits. Examples of impoverished perception show how experimenters can investigate these individual processes in a pure, but because of that, isolated and artificial way. In natural interactions with our environment we effortlessly overcome situations and events that should bring us to the boundaries of our capacity limitations – in a way that seems almost trivial to us. In this tutorial review we focus on selected literature that demonstrates how these limitations can be circumvented, often by taking a step towards the real-world. Our aim is to highlight how these goals can be achieved with the various examples from the literature that are discussed here.

## From “pure” to “messy” measures

Following the cognitive revolution of the 1950s, researchers within psychology, cognition, and neuroscience started to investigate so-called basic mental processes. Their aim was often to study them in a *pure* sense, without any interference from other processes, such as the goals we may have at a given moment, and the particular tasks or actions we perform (see, e.g., Baddeley & Hitch, 1974; Broadbent, 2004; Neisser, 1963, 1967; Sternberg, 1969; see review in Kristjánsson & Egeth, 2020). A popular way of assessing mechanisms of visual cognition was to break them down into fundamental operations measured with simple stimuli, stepping away from real-life conditions that could otherwise contaminate the purity of the measurements. One aim, for example, was to study the capacity of visual attention and visual memory (Neisser, 1967). These measurements have undeniably been successful as they have provided novel insights into fundamental cognitive mechanisms (Kristjánsson & Egeth, 2020).

While studies using sparse artificial stimuli and tasks that focus only on snapshots of behavior have provided a foundation for understanding visual attention and visual memory representations, we highlight that in order to understand the functional nature of visual attention, visual representations, and visual memory, it is crucial to also investigate their quality and detail within the realm of active natural behavior (Draschkow, Kallmayer, & Nobre, 2020; Foulsham, Walker, & Kingstone, 2011; Malcolm, Groen, & Baker, 2016; Tatler, 2014; Tatler & Land, 2011).

It is rare for us, for example, to make a concerted effort to explicitly remember our visual surroundings, such as the location of the plates when visiting our friends for dinner at their new flat. Tasks relying on explicit memorization procedures are nevertheless commonly used in studies within visual cognition. In real life, we more commonly complete goal-directed behavior, such as setting the table, during which the location and identity representation of the surrounding objects is generated “on the fly.” Recent work has shown that the representations generated through natural behavior are more reliable than those generated through explicit memorization (Draschkow, Wolfe, & Võ, 2014; Helbing, Draschkow, & Võ, 2020).

The well-known perceptual scientist James Gibson proposed the concept of “active perception,” claiming that perception can only be understood in the context of actions relevant to the stimuli and conditions in each given case (Gibson, 1966, 1979; Nakayama & James, 1994). Measuring the “pure” capacity of concepts such as memory or attention was therefore seen as meaningless unless such mechanisms are tested in the context that they have evolved for. An experimental approach that ignores context runs the risk of facing similar problems to those structuralists such as Wundt and Titchener faced over 100 years ago in their attempts at assessing elementary sensations: putative elementary sensations may simply not exist without a context (Leahey, 1981). A good example of Gibson’s approach is his “cookie cutter” experiment (Gibson, 1962). When a “cookie-cutter” was statically pushed onto participants’ palms, identification rates of its pattern were just under 50% (chance performance was 16.7%), while if the cutter was rotated both clockwise and counterclockwise in the observer’s palm, recognition accuracy increased to about 95%. The important point here is that with increased interaction with the stimulation, the measured resolution of haptic perception increased, or to use another phrase, its capacity increased. Shying away from the more naturalistic manipulation would have left us uniformed about the recognition capacity that is actually available during real-world behavior. This accords well with most literature reviewed here: In addition to the measurements of pure capacity, any assessment of basic abilities must in the end be applied to the context they are used for.

## Current goals and overview

Our aim in this tutorial review was to discuss selected recent research that demonstrates how the perceptual and cognitive limitations suggested by reductionistic approaches can be overcome with naturalistic stimuli and tasks. Our hope is that these examples can serve as templates from different domains that interested readers can use as inspiration for their own research.

The closer we get to natural behavior, the more we engage different effectors (such as eye or hand movements) and encounter perceptual mechanisms that are implicit, operating “for free” and “on the fly,” i.e., quickly, efficiently and effortlessly. An informative placeholder example of how real-world tasks affect conclusions about how we orient within the world (Hayhoe, 2017) comes from Land and Hayhoe (2001), who tested the relation of eye and hand movements as participants performed natural everyday tasks such as making a cup of tea or a sandwich. They found a strong interaction between reach and gaze; gaze usually landed on the next object in the action sequence before any signs of manual action. They concluded that eye movements are planned into the motor pattern and lead each action. This suggests that studying one process without taking the other into account may not provide a complete picture of our visual and motor behavior.

Similar considerations may also apply to studies of how we construct representations of the visual world. These representations may differ according to how we interact with the visual world in each case, which can have implications for what is typically called visual long-term memory (VLTM). In many studies within this field, observers are asked to perform tasks that are very different from what they experience during everyday life. As such, estimates of their capacity may not tell the whole story. We review how testing long-term memory in a “pure” manner – without embracing the complexities of natural contexts – may change the conclusions we draw about how VLTM representations are formed and used.

An important consideration is that we rarely perform individual cognitive operations in isolation. We do not perform an “object recognition” task when drinking our cup of tea, nor explicitly recognize a door handle before opening a door. We *use* these objects to enjoy a pleasant meeting with a friend or get away from an unpleasant one. Moreover, recognizing objects does not end our engagement with them. Complex cognitive operations such as visual search are a means to an end, as we will likely interact with the object we have searched for, and inversely their function also guides our search (Castelhano & Witherspoon, 2016). Interactions with our environment can strongly influence the representation of our visual space and should therefore be considered when the goal is to understand factors guiding behavior (Schütz-Bosbach & Prinz, 2007). A common dichotomy in visual cognition and visual neuroscience is that different pathways are involved in recognition versus action (Mishkin, Ungerleider, & Macko, 1983), which means that the intended perceptual or physical acts are highly important when trying to understand the mechanisms behind the action (Gross & Graziano, 1995; Maravita & Iriki, 2004; Perry & Fallah, 2017). Engel, Maye, Kurthen, and König (2013) postulated that perception should be studied as an “enactive” process, which involves ongoing interactions with our surroundings, and that our representations subserve our interactions with the world. These processes should not be studied outside their role in action generation.

## Capacity

A central concept in research of attention and visual memory is *capacity*, or, in other words, how much information those systems can process over a given time period, inspired by information theory (Shannon, 1948) and the concept of how many bits of information can be processed by a given system in a particular amount of time (Bush, 1936)*.*

Research within cognitive psychology over the last 60 years or longer has clearly demonstrated that attentional capacity is limited (Á. Kristjánsson & Egeth, 2020). But how limited is it? This is a notoriously difficult question, and the first observation is that this obviously depends on how the question is asked. To take one example, in so-called multiple-object-tracking tasks, observers are not able to track more than approximately four items without losing track of some of the items (Pylyshyn & Storm, 1988). Studies of visual search have also been used to estimate capacity. Attentional capacity has been thought to be essentially unlimited as long as a search target stands out from the distractors on a single feature (Treisman, Sykes, & Gelade, 1977; Wolfe & Horowitz, 2004). But if targets can only be distinguished by a conjunction of features, individual items need to be assembled by attention, and the search items need to be inspected one-by-one, as proposed in the well-known feature integration theory (Treisman & Gelade, 1980). Search slopes have been used as an index of attentional engagement in a search task, but this approach has limitations (Kristjánsson, 2015, 2016; see also Wolfe, 2016).

Another good example of how a simplification of real-world complexities has been used to assess visual attention involves *cueing* studies (Posner, Nissen, & Ogden, 1978), which have uncovered interesting dynamics such as the influence of symbolic versus exogenous cues (Jonides, 1981) or different temporal components of attentional orienting (Nakayama & Mackeben, 1989). These studies provide information about how quickly visual attention is drawn to suddenly appearing stimuli. It is of note that although real-world applications of these concepts can certainly be envisioned, studies of actual implementation in dynamic real-world environments are scarce.

## Attention and action

A theme in the current overview is that we should aim to also estimate the capacity of the mechanisms we use for interacting with the world with tasks that mimic our real-world interactions as closely as possible. When we interact with the world our hand and eye movements are *often* highly coupled, but this is not *always* the case: consider a musician reading music as she moves her fingers on a keyboard or a guitar neck, or a skilled typist, who types while keeping his gaze on the screen rather than on the keyboard. Are the attentional mechanisms used for eye and hand the same, or coupled? Coupling them can in some cases be useful when our goals for reach and gaze match, but this could be detrimental when they do not, as in the examples involving the musician and the typist. The study of attention over the last decades, however, has implicitly been investigated as a one-source concept – where gaze and reach are assumed to draw on the same resource.

Firstly, we should note that there is a lot of evidence that attention is tightly coupled to action (Deubel & Schneider, 1996; Kowler, Anderson, Dosher, & Blaser, 1995; Kristjánsson, 2011; Montagnini & Castet, 2007). Eye and hand movements have been shown to link visual attention to the endpoints of their actions during movement preparation (Deubel, Schneider, & Paprotta, 1998; Rolfs, Lawrence, & Carrasco, 2013). This has usually been measured with some assessment of discrimination performance (Hanning, Deubel, & Szinte, 2019a) at the intended endpoint of the movement. While the exact nature of this relationship has been debated, this relation is probably by no means a necessary one (Hanning, Deubel, & Szinte, 2019a; Kristjánsson, 2011; Van der Stigchel & de Vries, 2015; Wollenberg, Deubel, & Szinte, 2018), as has been assumed for example in the influential *premotor theory of attention* (Rizzolatti, Riggio, Dascola, & Umiltá, 1987). Effects of action preparation have been found when observers reach towards a target rather than moving their gaze towards it. Attentional performance is best just prior to the start of a reaching movement at the reach target (Baldauf & Deubel, 2010; Deubel et al., 1998; Rolfs et al., 2013). A number of authors have argued that the same attention mechanism is behind both the link between gaze and attention as well as reach and attention (Bekkering, Abrams, & Pratt, 1995; Huestegge & Adam, 2011; Khan, Song, & McPeek, 2011; Nissens & Fiehler, 2018; Song & McPeek, 2009).

Jonikaitis and Deubel (2011) asked observers to move their gaze or reach towards either the same or different locations. Surprisingly, they found that delaying eye movements delayed attention shifts to the gaze target without affecting attention at the locus of the reach target. In contrast with arguments for a unitary mechanism for selection, this suggests that eye and hand movements are selected by largely independent *effector*-*specific* attentional mechanisms. Even more importantly, their results suggest that the attentional benefit at one effector’s movement target is not affected by the concurrent movement preparation of the other effector to another location. There is evidence from single-cell neurophysiology (Graziano & Gross, 1996; Perry, Sergio, Crawford, & Fallah, 2015) and functional neuroimaging (Makin, Holmes, & Zohary, 2007) that visual processing can be enhanced when a reach places the hand near the stimuli to be processed. Perry and Fallah (2017) propose that visual processing near the perceived position of the hand is amplified because of feedback from frontoparietal areas. Since attention must often be divided between visual and motor tasks, other effector systems such as the reach/grasp system may also cause attention-related vision enhancement, which would undoubtedly be beneficial in many scenarios. Hanning, Aagten-Murphy, and Deubel (2018) then tested whether targets for eye movements and targets for reach movements are selected by the same attentional mechanism by measuring visual sensitivity – an established “proxy” for motor selection – at the motor targets during the preparation of simultaneous eye and hand movements. They found that sensitivity at both the eye and the hand target locations was unaffected by the simultaneous movement preparation of the other effector. Observers were able to allocate attention simultaneously to two different targets for a movement, arguing for separate attentional mechanisms for the two effector systems. Perhaps even more important was the finding that the two selection mechanisms did not seem to compete for resources at any point during the movement preparation process, at least when the necessary resources can be freed up from irrelevant locations (Kreyenmeier, Deubel, & Hanning, 2020. Hanning et al. argued that the gaze and reach targets are represented in effector specific maps, consistent with the neural evidence from Perry et al. (2015), who showed that when monkeys placed a hand close to a visual target, orientation tuning was sharpened, providing evidence for an effector-based mechanism that improves processing of features relevant to that effector (see also Graziano & Gross, 1996). While two attention systems can often appear as if they operate in unison, and such coupling may indeed be useful, they can also be dissociated (Graziano, 2001; Perry & Fallah, 2017). Consistent findings have been reported for patients with optic ataxia (Jackson, Newport, Mort, & Husain, 2005), where a patient’s deficit was confined to reach movements with his right hand. Zelinsky and Bisley (2015) have in fact pointed out that map-based representations are ubiquitous throughout the brain and that there are separate salience maps for different effectors. Despite evidence for separate attentional systems, the systems are often studied in isolation in standard laboratory-based tasks used for capacity estimates where observers’ body and eye movements are restricted. By using more complex tasks in which natural movements are not only allowed but also encouraged (Sauter, Stefani, & Mack, 2020), we may gain a fuller understanding of how our attentional systems cope with their own limitations.

## Attentional capacity during visual foraging

Recent evidence from foraging tasks argues that attentional capacity may be higher than proposed in prominent attention theories such as the influential feature integration (Treisman & Gelade, 1980) and guided search theories (Wolfe, 1994, 2007). These theoretical accounts are based on findings from visual search tasks where response times (RTs) for conjunction targets (where the target is distinguished from distractors by a conjunction of two features) increased linearly with each added item to the display, since attention was required to inspect each conjunction item (see exchange on this in Kristjánsson, 2015, 2016; Wolfe, 2016). Surprisingly, Kristjánsson, Jóhannesson, and Thornton (2014) found that during a foraging task where observers were asked to select many targets of two types (defined by a conjunction of features) on a tablet touch-screen, some observers switched easily between the target types with very low switch costs. This goes against a basic tenet of visual attention research from the last four decades or so: that attention is needed to perform a time-consuming integration of features for each object. Instead, this suggests that two conjunction templates could be simultaneously active (for replications, see Clarke, Irons, James, Leber, & Hunt, 2020; Wolfe, Cain, & Aizenman, 2019), or rapid switching between templates in working memory (WM) is possible (more on that in the *Foraging and visual working memory* section).

Further work then revealed that this was not necessarily confined to a subset of observers; when time limits (5, 10, or 15 s) were imposed on how long observers had to forage for as many targets as they could, most people increased their frequency of switching between different target types during conjunction-based foraging (Kristjánsson, 2018). This shows that under the appropriate task demands, performance can reach levels well above traditional capacity estimates from theoretical accounts inspired by the visual search literature. Capacity may be a more dynamic entity than often thought, and hand movements towards targets on tablet touch-screens reveal higher capacity than we would expect from traditional visual search studies. This is consistent with findings of higher attentional performance when the hands are near visual items that are used to assess attention (Abrams, Davoli, Du, Knapp, & Paull, 2008; Reed, Grubb, & Steele, 2006) and evidence that the visibility of the hands alters neural responses (Makin et al., 2007; Perry et al., 2015).

Note also that Kristjánsson, Thornton, Chetverikov, and Kristjánsson (2020b) showed that well-known set-size effects were only seen during selections of the last target during foraging and that selections preceding the last one were much faster. This demonstrates how our understanding of attentional mechanisms can change once we investigate it using a more interactive task – in this case finger foraging. We should note that proposals that there are separate neural mechanisms for hand and gaze selection (Makin et al., 2007; Perry et al., 2015) mean that finger foraging could differ from foraging with mouse movements (as tested, e.g., in Wolfe, 2013), but no study has directly compared finger foraging and mouse foraging within-participants.

Interestingly, the correlation in performance between gaze and finger foraging seems to be relatively low (Jóhannesson, Thornton, Smith, Chetverikov, & Kristjánsson, 2016; see also Tagu & Kristjánsson, 2020), and conjunction foraging is easier during gaze foraging, consistent with the proposal that the crucial mechanisms differ for gaze and finger selection, and that recruiting more effectors can help overcome limitations imposed by attention associated with a single effector. The constraints of the specific task at hand (Hayhoe & Rothkopf, 2011; Tatler, Hayhoe, Land, & Ballard, 2011) are critical and can even better account for observers’ attentional allocation compared to external factors, such as visual salience. Relatedly, Robinson, Benjamin, and Irwin (2020) found that estimates of capacity from different tasks may not overlap much and cannot be estimated by common parameters. This is an important point showing that overall capacity estimates may not always generalize well across task and context. We believe that the results summarized above suggest that the answers about capacity could depend on how the questions are asked.

## The capacity of visual working memory

Visual working memory (VWM) allows us to monitor our own mental representations and keep track of our goals as we interact with the visual world. Attention and VWM are strongly linked and share neural mechanisms to a considerable extent (Awh, Anllo-Vento, & Hillyard, 2000; Labar, Gitelman, Parrish, & Mesulam, 1999). Desimone and Duncan (1995) argued that WM elicits a neural signal that can bias selective attention, and in the Theory of Visual Attention (TVA) model (Bundesen & Habekost, 2012) our attentional goals are considered to be maintained in VWM.

In the past, VWM has often been studied with so-called change-detection tasks (e.g., Alvarez & Cavanagh, 2005; Luck & Vogel, 1997). Observers are shown an array of a number of visual items and asked to remember them. Shortly afterwards, following a blank screen or a mask, they are asked to judge whether a change occurred in the array or not. The aim with change-detection tasks has been to assess WM independently of other mechanisms. Another more recent approach to studying WM has involved *continuous reports* (Bays & Husain, 2008a; Zhang & Luck, 2008). There, instead of a change, a probe follows the mask and participants need to reproduce an item’s orientation, color, or location in a continuous fashion – for example, by using a color wheel. Estimates of the capacity of VWM have focused on a fixed number of items (Luck & Vogel, 1997) or a certain amount of information (Alvarez & Cavanagh, 2005; Bays & Husain, 2008).

But while these tasks emulate a world in which visual information rapidly changes and items disappear, our surroundings tend to remain rather stable across adjacent time points. The visual system is, in fact, notoriously bad at detecting changes (Simons & Levin, 1997; Simons & Rensink, 2005), perhaps because it makes strong assumptions about continuity (Chetverikov, Campana, & Kristjánsson, 2017b; Cicchini & Kristjánsson, 2016; Fischer & Whitney, 2014; Kristjánsson & Ásgeirsson, 2019).

## Motor action and visual working memory

VWM performance is influenced by our intended actions (van Ede, 2020). In Heuer, Crawford, and Schubö (2017), participants memorized a number of items and subsequently performed a pointing movement before their memory was tested at either the movement goal or an irrelevant location. Memory performance at intended movement goals was higher than at action-irrelevant locations, showing that like attention, visual memory can be bound to the actions we perform and to our goals.

Both eye movements (Bays & Husain, 2008b; Hanning, Jonikaitis, Deubel, & Szinte, 2016; Ohl & Rolfs, 2017) and hand movements (Hanning & Deubel, 2018; Heuer et al., 2017) have been found to enhance WM performance at their motor targets. This is not unexpected given that VWM and attention show strong overlap both functionally and in terms of neural mechanisms (Awh & Jonides, 2001; Jonikaitis & Moore, 2019). The findings of Hanning, Aagten-Murphy, and Deubel (2018) discussed above suggest that independent mechanisms drive attention to eye and hand targets. If this is true, this raises the question of whether the two effector systems also operate separately within WM. To address this, Hanning and Deubel (2018) asked their participants to memorize several locations. Participants had to either make *single* eye or hand movements or make simultaneous eye and hand movements to two distinct memorized locations. The authors found enhanced memory at the eye and hand motor targets, with no signs of any tradeoff between the two memory processes, for gaze and reach. This shows that WM at the saccadic goal and at the reach goal can be independent of one another, and that VWM can be augmented by effector specific memory.

Further, Chetverikov et al., (2018) found that untethering hand-guided and eye-guided attention improved WM performance. In another related finding, Hanning et al. (2016) found dissociable effects of task relevance and oculomotor selection on WM. They found that task relevance on its own, without the coupled oculomotor selection, did not lead to any improvement in WM, while oculomotor selection did. Effects of task relevance and oculomotor selection on WM performance for features could be separated, in other words. These results highlight the importance of studying how VWM operates in increasingly complex behavior.

## Foraging and visual working memory

Due to the close link between attention and WM, it is informative to revisit the foraging findings of Kristjánsson et al. (2014). During foraging for a given number of targets it is natural to assume that observer’s attention is guided by WM templates and foraging results can therefore cast light upon the operation of VWM. A recent proposal is that while VWM can contain more than one template, only one template is accessible for attentional guidance at any given moment (Olivers, Peters, Houtkamp, & Roelfsema, 2011; Ort, Fahrenfort, & Olivers, 2017; van Moorselaar, Gunseli, Theeuwes, & Olivers, 2014). The rapid switching between target types during feature foraging (described in the section on attention above) shows how such “one template at a time” limitations could be overcome in more natural scenarios. And the rapid switching during conjunction foraging (T. Kristjánsson, Thornton, & Kristjánsson, 2018) is even more informative, since these templates would require a complex exclusion rule based on two feature dimensions (shape and color) along with very fast feature integration, yet observers seem to be able to do this. This raises the intriguing question of whether the two non-overlapping attentional systems that Hanning and Deubel (2018) found evidence for allow for higher capacity performance than the tasks used, for example, by van Moorselaar, Gunseli, Theeuwes, and Olivers (2014), since the foraging task involves concurrent gaze and finger selection. As mentioned above, Kristjánsson et al. (2018) found that as observers were told to collect as many conjunction targets as they could, they were actually able to switch between target types rapidly, but during tasks where they had unlimited time to forage for conjunction targets they seemed to avoid switching. Kristjánsson et al. (2018) speculated that this showed that observers could load VWM with more information but that they preferred to avoid this because of the effort involved (Thornton, Nguyen, & Kristjansson, 2020), and would therefore particularly avoid this during longer duration tasks. That most observers could rapidly switch between conjunction targets when needeed strongly supports that WM capacity is flexible, interacting with task demands. In further investigations, Thornton, de’Sperati, and Kristjánsson, Ólafsdóttir, and Kristjánsson (2019) used three different selection methods during a foraging task, finding that observers switch very frequently even during conjunction foraging, suggesting that observers can load WM with two complex templates simultaneously. Notably, they used both moving and static displays and, interestingly, the tendency to switch categories typically increased when targets moved, suggesting that increased attentional demands from motion do not necessarily induce larger run numbers during foraging, which would presumably reflect greater WM demands. We also note that similar results have now been reported for foraging in virtual reality displays, making the connection with more complex and realistic tasks even stronger (Kristjánsson, Draschkow, Pálsson, Haraldsson, Jónsson, & Kristjánsson, 2020a).

Kristjánsson and Kristjánsson et al. (2018) then tested a foraging task where they varied the number of targets and distractors. They found that switch costs increased roughly linearly with the size of the memory set, consistent with load accounts of VWM. Again, this shows how active tasks can inform theoretical accounts. Foraging studies have also been used to shed new light on the development of WM, along with other executive functions (Ólafsdóttir, Gestsdóttir, & Kristjánsson, 2019, 2020).

We want to emphasize that active tasks can change how we think about VWM. Ballard, Hayhoe, Li, and Whitehead (Ballard, Hayhoe, Li, & Whitehead, 1992) asked their participants to reproduce an array of colored blocks in a certain model arrangement that was visible in an adjacent panel, picking blocks from an available pile. They expected observers to take a look at the model area, memorize the position and color of the blocks, and then place the blocks in the copy area. But instead they found that observers continually checked back and forth from the model to the copy area. In other words, they did not seem to memorize the whole area but only a small amount of information at a time. This highlights that while VWM capacity might be much higher than many estimates have posited, under more natural circumstances observers may choose to not rely on these resources (Ballard, Hayhoe, & Pelz, 1995; Draschkow et al., 2020). This general performance pattern has also been seen in the foraging literature where observers are less likely to switch targets when given more time to forage within displays (see review in Kristjánsson et al., 2019).

## The role of memory

To overcome the limits of capacity-restricted cognitive functions we can incorporate expectations and knowledge about our current behavioral context. These priors can come from different time scales (Nobre & Stokes, 2019) – from the immediate past, such as from priming, from long-term episodic and semantic representations, or from memories from an intermediate time scale, such as trial history or serial dependence effects. In this section we review some selected exemplar literature that highlights how prior knowledge can support attention and WM.

## The recent past supports attention and working memory

The visual system relies on recent representations to construct current percepts as shown for example in serial dependence (J. Fischer & Whitney, 2014; Manassi, Liberman, Chaney, & Whitney, 2017; Manassi, Kristjánsson, & Whitney, 2019; Pascucci et al., 2019). Contextual information supports the integration of the recent past and present in order to enable stable percepts across time (C. Fischer et al., 2020). Critically, these recent experiences do not simply alter but can also *facilitate* perception (Cicchini, Mikellidou, & Burr, 2018). Studies of so-called feature-distribution learning (Chetverikov, Campana, & Kristjánsson, 2016, Chetverikov, Campana, & Kristjánsson, 2017c; Hansmann-Roth, Chetverikov, & Kristjánsson, Hansmann-Roth, Chetverikov, & Kristjánsson, 2019; Rafiei, Hansmann-Roth, Whitney, Kristjánsson, & Chetverikov, 2020) show that we can encode abstract details of preceding information in the environment. Priming effects in vision (Ásgeirsson, Kristjánsson, & Bundesen, 2015; Brascamp, Blake, & Kristjánsson, 2011; Maljkovic & Nakayama, 1994; for a review, see Á. Kristjánsson & Ásgeirsson, 2019) show that attention deployments are strongly determined by perceptual history. These insights highlight that we perform tasks within a temporal context and this context will in turn influence the estimates of capacity. That is, attention does not operate in a vacuum – the current event context is highly important. Curiously, the size of priming effects dwarfs many other effects such as effects of top-down attention (Á. Kristjánsson & Ásgeirsson, 2019; Kristjánsson, Wang, & Nakayama, 2002; Maljkovic & Nakayama, 1994; for a review, see Theeuwes, 2013).

Similar to attentional performance, VWM is influenced by recent experiential history (Carlisle & Kristjánsson, 2018; Cochrane, Nwabuike, Thomson, & Milliken, 2018; Cunningham & Egeth, 2016; Kristjánsson, Saevarsson, & Driver, 2013). For example, Carlisle and Kristjánsson et al. (2018) showed how priming and WM can affect one another and argued that implicit short-term memory and explicit VWM interact when they provide conflicting attentional instructions. Further, WM capacity is usually estimated by repeating the same or very similar stimuli between all trials of an experiment. This can lead to interference between consecutive trials, and in fact capacity estimates are considerably larger when such proactive interference is discouraged by using unique stimuli (Endress & Potter, 2014; Hartshorne, 2008).

## Long-term representations support attention and working memory

There is rich evidence that long-term semantic (Henderson & Hayes, 2017; Torralba, Oliva, Castelhano, & Henderson, 2006; Võ & Henderson, 2010; Võ & Wolfe, 2015; Wolfe, Võ, Evans, & Greene, 2011) and episodic (Aly & Turk-Browne, 2017; Brockmole & Henderson, 2006; Chun & Jiang, 1998, 1999, 2003; Draschkow & Võ, 2016, 2017; Fan & Turk-Browne, 2016; Hutchinson & Turk-Browne, 2012; Patai, Buckley, & Nobre, 2013; Stokes, Atherton, Patai, & Nobre, 2012; Summerfield, Lepsien, Gitelman, Mesulam, & Nobre, 2006; Võ & Wolfe, 2012) memory representations support the allocation of attention. These long-term representations are critical in enabling a seamless and continuous visual experience, because in order to overcome limitations in capacity, long-term priors extracted within the initial glimpse of an environment (Oliva, 2005) can provide clues about target appearance (Robbins & Hout, 2019) and guide attention to the most informative locations (Võ, Boettcher, & Draschkow, 2019; Wolfe et al., 2011).

With regard to WM, a striking example of how interactions between long- and short-term representation can overcome classical WM capacity limits is provided by Endress and Potter (2014). They tested capacity for briefly presented *familiar* objects (at 4 or 8 Hz) with probes following the stream of stimuli, finding very high WM capacity (up to 30 items) when all the objects were *unique* throughout the experiment (avoiding proactive interference), while if the items were recycled across trials within the experiment, capacity estimates were much lower. Since many WM experiments depend on repeating stimuli (such as colored squares, oriented bars, or locations on the screen), this finding suggests that proactive interference may explain at least some of the limits in WM capacity traditionally found in the literature.

Brady and Störmer (2020) measured VWM with real-world objects and stripped-down single-feature colored stimuli. Their results suggest that VWM performance depends highly on whether single feature objects are used or whether real-world objects are used – not only did they find the benefit for meaningful objects but also the real-world objects benefitted from sequential presentation, which the colored patches did not – suggesting that the encoding for the two different stimuli-types may differ (Brady, Störmer, & Alvarez, 2016).

Finally, hybrid search tasks have demonstrated the remarkable efficiency of searching through visual space for any one of up to 100 targets held in memory (Wolfe, 2012). In these tasks, participants memorize upwards of 100 objects during a learning session and subsequently perform visual searches for these items amongst visual arrays of novel distractor objects. While visual search times increase linearly with an increase in items in the visual display, searching within memory for visual familiar objects (Wolfe, 2012) or words (Boettcher & Wolfe, 2015) increases logarithmically. Critically, searching for the groceries on your shopping list does not require you to search through the entire supermarket for as many times as the number of items on your list. Instead, you go through it once and perform many memory searches “on the fly” (Drew, Boettcher, & Wolfe, 2017). So, while WM memory might have limited capacity for holding the attentional template that is relevant to the current search, hybrid search results demonstrate that we can search for a number of targets with astonishing efficiency, even more so for if the objects of our search are familiar, instead of novel (Madrid, Cunningham, Robbins, & Hout, 2019).

## Building and using behaviorally optimal long-term representations

While the earlier sections focused on cognitive systems which are renowned for their limitations, visual long-term memory (VLTM) is famously boundless. Early studies of VLTM showed remarkably large storage capacity as observers could determine if they had seen one of two images with over 80% accuracy even after viewing 10,000 scenes (Standing, 1973). In addition to capacity, studies have provided evidence for high VLTM detail (Brady, Konkle, Alvarez, & Oliva, 2008; Cunningham, Yassa, & Egeth, 2015; Draschkow et al., 2014; Konkle, Brady, Alvarez, & Oliva, 2010a, 2010b) and longevity (Hollingworth, 2004, 2006; Hollingworth & Henderson, 2002; Konkle et al., 2010b). Quite remarkably, even when related studies with alternative retrieval tests indicated more modest VLTM capacity, there was still no significant drop in the detail of existing representations (Cunningham et al., 2015).

In the previous section we highlighted that long-term representations are critical for the efficient guidance of attention and WM. While there is debate about the representational format of this vast storage of information, here we turn to the question of how the visual system utilizes and forms VLTMs – as not all representations are built in the same way. Memory representations of our surroundings are closely determined by what we have seen, but also by what we have attended. Memory performance is, for example, predicted by how long (Hollingworth & Henderson, 2002) and how often we fixate an object (Tatler, Gilchrist, & Land, 2005; Tatler & Tatler, 2013). For the current topic, it is important to note that task-relevant objects are remembered better than irrelevant ones (Castelhano & Henderson, 2005; Maxcey-Richard & Hollingworth, 2013; Williams, Henderson, & Zacks, 2005) and memory representations strongly interact with behavioral goals (Droll & Hayhoe, 2007; Droll, Hayhoe, Triesch, & Sullivan, 2005; Triesch, Ballard, Hayhoe, & Sullivan, 2003), which becomes particularly evident in natural behavior (Tatler & Land, 2011).

In parallel with the previous sections, we emphasize the importance of studying active natural behavior (Draschkow et al., 2020; Foulsham et al., 2011; Malcolm et al., 2016; Tatler, 2014; Tatler et al., 2011) and how VLTMs are generated as a natural by-product of interactions with the environment (Draschkow & Võ, 2017; Helbing et al., 2020), as these representations support seamless everyday activities. In comparison to memory investigations in which *memorization* is the explicit task, during ecological behavior it is not necessary to constantly instruct ourselves to remember everything in our surroundings. In fact, an ever-growing body of literature provides strong evidence that very reliable representations are formed after incidental encoding during search (Castelhano & Henderson, 2005; Draschkow et al., 2014; Draschkow & Võ, 2016; Hout & Goldinger, 2010, 2012; Howard, Pharaon, Körner, Smith, & Gilchrist, 2011; Olejarczyk, Luke, & Henderson, 2014; Võ & Wolfe, 2012), change detection (Utochkin & Wolfe, 2018), visual discrimination (Draschkow, Reinecke, Cunningham, & Võ, 2018), or object manipulation (Draschkow & Võ, 2017; Kirtley & Tatler, 2015). Draschkow et al. (2018) investigated the capacity and detail of incidental memory, instructing participants to detect visually distorted objects among a stream of intact objects (the incidental analogue to the explicit studies of Brady et al., 2008, and Cunningham et al., 2015). In a subsequent surprise recognition memory test, they found that even after very brief exposures to thousands of isolated objects, incidental memory was above chance. Another example of incidental memory being more robust than one might intuitively assume is Pinto, Papesh, and Hout’s (2020) visual search study. They employed a challenging surprise memory test that probed incidental object representations by showing participants up to as many as 16 possible alternatives (e.g., “which of these 16 butterflies did you see while searching?”). Using a quantification of object similarity via multidimensional scaling ratings, the study provides evidence that even under very adverse conditions perceptual details are being retained following incidental encoding.

Not only do we seem to be able to generate strong incidental representations, but the memories we have gathered on the fly, during natural interactions, might in fact be critical for proactively guiding our behavior. Chetverikov, Campana, and Kristjánsson (2017a) have shown how repeated searching within search arrays with particular feature distributions of orientation or color (Chetverikov et al., 2017c; Tanrikulu, Chetverikov, & Kristjánsson, 2020) enables observers to learn the probabilities of feature values and build up a probabilistic template of the set for distractor rejection (Chetverikov, Campana, & Kristjánsson, 2020a). Using a repeated-search task, Võ and Wolfe (2012) demonstrated that attentional guidance by memories from previous encounters was more effective if these memories were established when *looking for* an item (during search), compared to *looking at* targets (explicit memorization and free viewing). The task at hand is critical for the information that gets extracted from fixations in real-world environments (Tatler et al., 2013). Further, search for objects is speeded if these objects have been incidentally fixated on preceding trials both in real (Draschkow & Võ, 2016) and virtual (Draschkow & Võ, 2017) environments.

The more naturalistic a task becomes; the more incidental representations gain strength. Object handling improves the speed of subsequent object recognition over passively viewed objects (Harman, Humphrey, & Goodale, 1999; James et al., 2002). Locations were recalled better when participants made active hand movements to them compared to when the hand was passively moved (Trewartha, Case, & Flanagan, 2015). Search within naturalistic images created more robust memories for the identity of target objects than representations formed as a result of explicit memorization (Draschkow et al., 2014; Josephs, Draschkow, Wolfe, & Võ, 2016). During immersive searches in virtual reality this *search superiority* even leads to more reliable incidentally generated spatial representations when compared to memories formed under explicit instruction to memorize (Helbing et al., 2020). Critically, incidental encoding seems to strongly rely on the availability of meaningful scene semantics in the stimulus materials used (Draschkow et al., 2014; Võ et al., 2019). The search superiority effect is diminished when no semantic contextual information is provided (Draschkow et al., 2014) or participants are not given enough time to associate the context with the target (Josephs, Draschkow, Wolfe, & Võ, 2016), although it is of note that the memory representations of items searched for was no worse than those explicitly memorized even in the absence of scene semantics.

In natural behavior, new information is easily integrated with prior knowledge, as we rarely encounter items that are “new enough” to require integration effort. It is thus sensible to incorporate this information incidentally and on the fly, instead of trying to “force” new memories in explicitly. Virtual reality paves the way for studies in realistic and unconstrained task settings that can probe such dynamics, while maintaining a high degree of experimental control (David, Beitner, & Võ, 2020; Draschkow et al., 2020; Draschkow & Võ, 2017; Figueroa, Arellano, & Calinisan, 2018; Kit et al., 2014; Li, Aivar, Kit, Tong, & Hayhoe, 2016; Li, Aivar, Tong, & Hayhoe, 2018; Olk, Dinu, Zielinski, & Kopper, 2018).

## Summary and general conclusions

While reductionist approaches are a cornerstone of empirical research in cognition, there are definite limits to studying real-world vision in artificially stripped-down settings. Our aim was to review selected recent findings that showcase how the basic mechanisms of visual attention and working and long-term memory operate within a framework that embraces various real-world complexities, and to highlight how such real-world paradigms can be used to inform our ideas about visual cognition.

## Implications for visual attention and working memory

The evidence presented here suggests that attention may in fact be intrinsically bound to the involved effectors, as indicated by the results of dissociating reach and gaze (Chetverikov et al., 2018; Hanning et al., 2016) as well as supporting neural evidence (Gross & Graziano, 1995; Perry & Fallah, 2017). It is important to note that our aim with this claim is not to restate the well-known premotor theory of attention (Rizzolatti et al., 1987). Our claim is simply that any “pure” measurements of capacity may not encapsulate how we attend (in a general sense) in more natural tasks. Results from visual foraging tasks (Kristjánsson et al., 2014; 2020) indicate that capacity limitations can be flexibly circumvented (Kristjánsson et al., 2018; Thornton et al., 2020), and that, if needed, observers seem to behave as if they have higher capacity, but only when this is necessary due to task constraints – perhaps because of the effort involved in loading WM (Ballard et al., 1995; Draschkow et al., 2020).

We also note that attention has been thought to operate on priority maps (Itti & Koch, 2001; Koch & Ullman, 1985). Our review raises the possibility that different priority maps may exist for different action effectors, as argued by Zelinsky and Bisley (2015). This is also in line with the claims of Perry and Fallah (2017) that there are specific attentional mechanisms for each effector (such as the eye vs. the hand). Note that if separate priority maps do indeed exist, this would make the idea of pure context-free capacity suspect if each effector has its own attentional prioritization mechanism. This would, on the other hand, allow considerable flexibility. Such arrangements could, for example, enable our musician from the *Introduction* to efficiently move her fingers across the piano keys while she independently keeps her gaze on the sheet music in front of her.

Considering the limitations in WM capacity, it is highly interesting that during combined eye and hand movements, memory can be improved at two different locations simultaneously at little or no cost (Chetverikov et al., 2018; Hanning & Deubel, 2018). In other words, WM capacity is higher when two effectors are simultaneously recruited. This calls for a different conception of WM that includes the actions we perform (Myers, Stokes, & Nobre, 2017; van Ede, 2020), and argues that putative capacity estimates need careful consideration when they are extended to natural behavior.

Finally, the limitations of attention and WM can often be overcome by incorporating representations from the recent and distant past (Nobre & Stokes, 2019). Reducing the proactive interference in the experimental approach can substantially improve estimates of capacity (Endress & Potter, 2014; Hartshorne, 2008). Testing WM performance with real objects strongly improves performance, and therefore capacity (Brady & Störmer, 2020; Endress & Potter, 2014). We also note that we can search for hundreds of targets with astonishing efficiency (Boettcher & Wolfe, 2015; Wolfe, 2012). That is, attention and WM do not operate in a vacuum, and investigations of how the past supports the future are important for our understanding of how we perform tasks efficiently despite the limits of “pure” attention and WM.

## Implications for long-term memory

Natural tasks in screen-based, real or virtual settings can reveal how long-term memory representations are formed via implicit learning mechanisms. A surprising amount of information about the environment may be picked up for free (Castelhano & Henderson, 2005; Williams, 2010). Moreover, incidental memories generated through natural behavior may be more robust than those picked up during explicit memorization (Draschkow et al., 2014; Helbing et al., 2020; Josephs et al., 2016; Võ & Wolfe, 2012). In other words, when people interact with the environment, more information is accumulated than when people perform more artificial memory tasks. This highlights the importance of understanding more natural encoding conditions, as they might deviate from estimates from traditional tasks.

How might these long-term memory representations – which are so important for guiding behavior – be formed? One clue may come from recent studies of so-called feature distribution learning (for a review, see Chetverikov, Hansmann-Roth, Tanrıkulu, & Kristjánsson, 2020b). Chetverikov and colleagues (Chetverikov, Campana, & Kristjánsson, 2016, 2017a) used a novel method to investigate whether observers can encode the shape of a probability density function of distractor distributions in odd-one-out visual search tasks for orientation and color. Instead of using explicit judgments of distribution statistics, they measured observers’ visual search times, which revealed observers’ expectations of distractor distributions. They used slowing effects from role-reversals between the target and distractors, which occur when feature values of target and distractors used on previous search trials are swapped on the next (Á. Kristjánsson & Driver, 2008; Lamy, Antebi, Aviani, & Carmel, 2008; Wang, Kristjansson, & Nakayama, 2005). They found that observers were able to encode the statistics of feature distributions in surprising detail – much more detail than previous studies have indicated (Alvarez, 2011; Cohen, Dennett, & Kanwisher, 2016). Critically, this learning is implicit, and does not require the explicit report of the properties of the stimuli, but can nevertheless guide action (Hansmann-Roth et al., 2020). Testing such feature distribution learning in more realistic settings, including virtual reality environments, might therefore be of great value in future.

## Conclusions

In this tutorial review we have provided several examples of how capacity limitations in visual cognition are overcome when attention, action, and memory cooperate, and have attempted to give examples of how such studies were implemented. Attention and memory may be intrinsically bound to the involved effectors, and our discussion highlights how long-term representations can provide the framework in which limitations might go unnoticed. Finally, natural tasks can establish representations incidentally, which subsequently become usable for proactive guidance. Taken together, we highlight the importance of investigating basic cognitive mechanisms as they unfold in increasingly complex behavior.

Note that we do not wish to claim that pure measurements are in any sense wrong – they have led to milestone discoveries concerning visual cognition. But measurements from such approaches should preferably be made to pass the real-world test; shown to apply to real-world settings as soon as possible. Yet a troubling possibility is that what we have called “pure” capacity measurements have little practical application. In some cases, they *may not exist* outside the paradigms that are used to measure them. This conclusion is probably unnecessarily pessimistic, however. A more constructive one could be that findings from reductionistic approaches generalize well, but concepts about visual mechanisms should be tested in more naturalistic conditions involving stimuli and tasks that do justice to the actual complexity of natural interactions.
